# Progress Toward Achieving and Sustaining Maternal and Neonatal Tetanus Elimination — Worldwide, 2000–2020

**DOI:** 10.15585/mmwr.mm7111a2

**Published:** 2022-03-18

**Authors:** Florence A. Kanu, Nasir Yusuf, Modibo Kassogue, Bilal Ahmed, Rania A. Tohme

**Affiliations:** ^1^Global Immunization Division, Center for Global Health, CDC; ^2^Immunization, Vaccines and Biologicals, World Health Organization, Geneva, Switzerland; ^3^Maternal, Newborn, and Adolescent Health Program Division, UNICEF, New York, New York.

Maternal and neonatal tetanus (MNT)* remains a major cause of neonatal mortality with an 80%–100% case-fatality rate among insufficiently vaccinated mothers after unhygienic deliveries, especially in low-income countries (*1*). In 1989, the World Health Assembly endorsed elimination^†^ of neonatal tetanus; the activity was relaunched in 1999 as the MNT elimination (MNTE)^§^ initiative, targeting 59^¶^ priority countries. MNTE strategies include 1) achieving ≥80% coverage with ≥2 doses of tetanus toxoid–containing vaccine (TTCV2+)** among women of reproductive age through routine and supplementary immunization activities (SIAs)^††^ in high-risk districts,^§§^ 2) achieving ≥70% of deliveries by a skilled birth attendant,^¶¶^ and 3) implementing neonatal tetanus case-based surveillance (*2*). This report summarizes progress toward achieving and sustaining MNTE during 2000–2020 and updates a previous report (*3*). By December 2020, 52 (88%) of 59 priority countries had conducted TTCV SIAs. Globally, infants protected at birth*** against tetanus increased from 74% (2000) to 86% (2020), and deliveries assisted by a skilled birth attendant increased from 64% (2000–2006) to 83% (2014–2020). Reported neonatal tetanus cases worldwide decreased by 88%, from 17,935 (2000) to 2,229 (2020), and estimated deaths decreased by 92%, from 170,829 (2000) to 14,230 (2019).^†††^ By December 2020, 47 (80%) of 59 priority countries were validated to have achieved MNTE, five of which conducted postvalidation assessments.^§§§^ To achieve elimination in the 12 remaining countries and sustain elimination, innovation is needed, including integrating SIAs to cover multiple vaccine preventable diseases and implementing TTCV life course vaccination.

## Immunization Activities

To estimate TTCV vaccination coverage delivered through routine immunization services and the number of neonates protected at birth from tetanus, World Health Organization (WHO) and UNICEF use data from administrative records and vaccination coverage surveys reported annually by member countries (*4*). WHO and UNICEF receive summaries of the number of women of reproductive age receiving TTCV during SIAs (*5*). In 2020, 16 (27%) of 59 priority countries achieved ≥80% TTCV2+ coverage, with 34 countries increasing coverage since 2000 (Table). In 2020, among 58 priority countries with available data, 46 (79%) reported ≥80% of infants protected at birth. The global proportion of infants protected at birth increased from 74% (2000) to 86% (2020) (Table).

**TABLE:** Indicators of maternal and neonatal tetanus elimination — 59 priority countries, 2000–2020

Country	≥2 TTCV doses among women of reproductive age* (%)			Newborns protected at birth (%)			Women of reproductive age vaccinated during TTCV SIAs		Skilled birth attendant at delivery^†^ (%)			No. of neonatal tetanus cases		
	Year		Change 2000–2020 (%)	Year		Change 2000–2020 (%)	No. of TT2+/Td2+ doses received	Vaccinated (%)	Year		Change 2000–2020 (%)	Year		Change 2000–2020 (%)
	2000	2020		2000	2020				2000^†^	2020^†^		2000	2020	
**Validated for maternal and neonatal tetanus elimination by end of 2020**														
Bangladesh	89	94	6	89	98	10	1,438,374	47	12	59	388	376	41	−89
Benin	81	83	2	87	81	−7	1,399,461	97	66	78	19	52	27	−48
Burkina Faso^§^	NA	69	NA	57	95	67	2,306,835	91	38	80	111	22	5	−77
Burma	81	83	3	79	90	14	8,170,763	87	57	60	6	41	17	−59
Burundi	28	89	218	51	90	76	679,222	55	25	85	238	16	0	−100
Cambodia	40	77	92	58	95	64	2,099,471	79	32	89	180	295	7	−98
Cameroon	40	62	56	54	83	54	2,687,461	85	56	69	23	279	16	−94
Chad	12	74	520	39	78	100	3,222,840	84	14	24	77	142	251	77
China	NA	NA	NA	NA	NA	NA	NA	NA	97	100	3	3,230	32	−99
Comoros	40	78	95	57	83	46	160,767	55	62	NA	NA	NA	0	NA
Congo	39	72	85	67	87	30	273,003	91	83	91	9	2	54	2,600^¶^
Côte d’Ivoire	78	75	−3	76	86	13	5,924,527	85	63	74	17	30	17	−43
Democratic Republic of the Congo	25	96	283	45	85	89	10,342,937	92	61	85	40	77	48	−38
Egypt	71	NA	NA	80	86	8	2,518,802	87	61	92	50	321	2	−99
Equatorial Guinea	30	36	20	61	60	−2	26,466	9	65	NA	NA	NA	4	NA
Eritrea	25	65	160	80	99	24	NA	NA	28	NA	NA	4	0	−100
Ethiopia	32	90	181	54	90	67	13,210,107	84	6	50	789	20	45	125
Gabon	16	43	171	39	83	113	79,343	90	86	NA	NA	8	1	−88
Ghana	73	62	−15	69	90	30	1,666,666	87	47	79	68	80	0	−100
Guinea-Bissau	NA	90	NA	49	83	69	312,669	98	32	54	69	NA	3	NA
Haiti	NA	44	NA	41	80	95	2,785,588	88	24	42	75	40	4	−90
India	80	78	−2	85	90	6	7,643,440	94	43	81	92	3,287	162	−95
Indonesia^§^	81	54	−34	82	85	4	1,442,264	50	66	95	43	466	4	−99
Iraq	55	42	−24	75	73	−3	111,721	96	65	96	47	37	0	−100
Kenya	51	NA	NA	68	88	29	4,463,695	67	43	70	65	1,278	0	−100
Laos	45	40	−12	58	93	60	968,323	90	17	64	286	21	12	−43
Liberia	25	20	−18	51	90	76	288,984	57	51	84	66	152	1	−99
Madagascar	40	52	30	58	75	29	2,705,588	72	47	46	−2	13	42	223
Malawi	61	70	15	84	90	7	NA	NA	56	90	62	12	NA	NA
Mauritania	NA	31	NA	44	83	89	586,277	76	53	69	30	NA	0	NA
Mozambique^§^	61	88	45	75	86	15	605,640	79	48	73	53	42	155	269
Namibia^§^	60	96	60	74	90	22	NA	NA	76	NA	NA	10	NA	NA
Nepal	60	80	33	67	89	33	4,537,864	86	12	77	549	134	3	−98
Niger	31	79	155	63	83	32	2,184,277	92	16	39	149	55	1	−98
Philippines	58	39	−33	55	91	65	1,034,080	78	58	84	46	281	28	−90
Rwanda^§^	NA	70	NA	81	97	20	NA	NA	31	94	201	5	0	−100
Senegal	45	68	51	62	95	53	359,845	92	58	75	29	0	0	NA
Sierra Leone	20	95	377	53	93	75	1,704,814	102	37	87	134	36	7	−81
South Africa	65	NA	NA	68	90	32	NA	NA	91	97	6	11	3	−73
Tanzania	77	92	19	79	91	15	987,575	71	43	64	46	48	2	−96
Timor-Leste	NA	69	NA	NA	83	NA	24,141	53	24	57	136	NA	2	NA
Togo	47	71	52	63	83	32	262,130	87	35	69	96	33	12	−64
Turkey	36	67	85	50	95	90	1,242,674	58	83	97	17	26	0	−100
Uganda	42	65	54	70	83	19	2,448,527	86	36	74	106	470	35	−93
Vietnam^§^	90	88	−2	86	96	12	367,842	69	59	NA	NA	142	41	−71
Zambia	61	NA	NA	78	85	9	330,030	81	42	80	91	130	26	−80
Zimbabwe	60	62	4	76	87	14	NA	NA	NA	86	NA	16	1	−94
**Not validated for maternal and neonatal tetanus elimination by end of 2020**														
Afghanistan	20	82	308	32	63	97	5,212,394	45	14	59	311	139	NA	NA
Angola	NA	41	NA	60	70	17	7,097,552	84	NA	50	NA	131	156	19
Central African Republic	20	88	341	36	63	75	804,984	30	32	40	27	37	177	378
Guinea	43	84	95	79	83	5	4,773,787	55	49	55	14	245	63	−74
Mali	62	39	−37	50	87	74	4,158,201	49	41	67	66	73	8	−89
Nigeria	NA	32	NA	57	65	14	9,365,295	66	35	43	23	1,643	55	−97
Pakistan	51	62	22	71	85	20	25,405,510	84	23	71	209	1,380	504	−63
Papua New Guinea	10	32	219	24	67	179	450,739	15	39	56	45	138	4	−97
Somalia	22	66	200	47	60	28	497,561	27	19	32	65	966	NA	NA
South Sudan	NA	61	NA	NA	65	NA	6,002,402	64	NA	NA	NA	NA	3	NA
Sudan	34	49	43	61	81	33	7,365,615	86	NA	NA	NA	88	34	−61
Yemen	31	22	−30	54	70	30	3,546,356	53	27	NA	NA	174	91	−48

**Sources: **Neonatal tetanus data: WHO Global Health Observatory Data Repository (2000–2020), Protected at birth data: WHO/UNICEF Joint Reporting Form on Immunization (2000–2020), Skilled birth attendant data: WHO Global Health Observatory Data Repository (2000–2020), SIA data: WHO/UNICEF Maternal and Neonatal Tetanus Elimination Database, as of January 2022, TTCV data: WHO Global Health Observatory Data Repository (2000–2020).

**Abbreviations:** NA = not available; SIA = supplementary immunization activity; TT2+/Td2+ = ≥2 doses of tetanus toxoid/tetanus-diphtheria toxoid; TTCV = tetanus toxoid–containing vaccine; WHO = World Health Organization.

* Includes first-year SIA conducted in Bangladesh in 1999 and first- and second-year SIAs conducted in Ethiopia in 1999.

^†^ Includes skilled birth attendant surveys conducted within 5 years for year 2000 and year 2020.

^§^ Administrative data of TTCV coverage with ≥2 doses among women of reproductive age were used when official data were unavailable for select country.

^¶ ^The increase in neonatal tetanus cases seen from 2000 to 2020 might be the result of improvement in surveillance.

**FIGURE 1:**
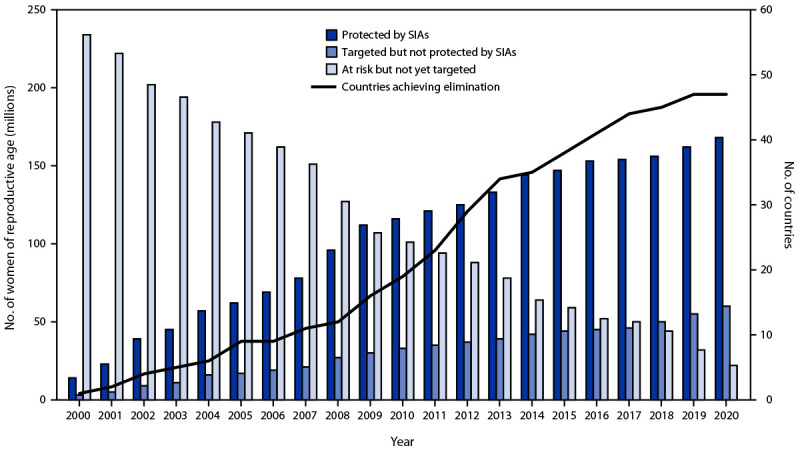
Number of women of reproductive age protected by tetanus toxoid–containing vaccine* received during supplementary immunization activities, number targeted but not yet vaccinated, number not yet targeted, and number of priority countries achieving maternal and neonatal tetanus elimination — worldwide, 2000–2020 **Source:** WHO/UNICEF Maternal and Neonatal Tetanus Elimination Database, as of January 2022. **Abbreviations:** SIA = supplementary immunization activities; WHO = World Health Organization. * Protected with 2 doses of tetanus toxoid or 2 doses of tetanus and diphtheria toxoids.

During 2000–2020, 52 priority countries conducted TTCV SIAs, and 168 million (67%) of the targeted 250 million women of reproductive age received TTCV2+ (Table) (Figure 1). In 2020, 59 million women targeted for protection by TTCV SIAs remained unreached, and TTCV SIA activities aiming to target an estimated 16 million women of reproductive age in five countries were postponed because of COVID-19–related disruptions in immunization services (Figure 1) (*6*).

## Deliveries Assisted by Skilled Birth Attendants

WHO and UNICEF estimate the percentage of births assisted by a skilled birth attendant from health care facility reports and coverage survey estimates shared by countries (*7*). During 2000–2020, the percentage of deliveries assisted by a skilled birth attendant increased 30%, from 64% (2000–2006) to 83% (2014–2020) (*7*). In 2020, among 50 priority countries with available data, ≥70% of deliveries were assisted by a skilled birth attendant in 28 (58%) countries (Table).

## Surveillance Activities

WHO recommends nationwide, case-based surveillance for neonatal tetanus, including zero-case reporting (submission of reports even if no neonatal tetanus cases are observed) and active surveillance through regular site visits (*8*). The number of reported neonatal tetanus cases worldwide decreased by 88% from 17,935 (2000) to 2,229 (2020).^¶¶¶^ In 2020, among all 59 priority countries, 10 (17%) reported zero cases, whereas seven countries (Angola, Central African Republic, Chad, Congo, Ethiopia, Madagascar, and Mozambique) reported more cases in 2020 than in 2000 (Table).

**FIGURE 2:**
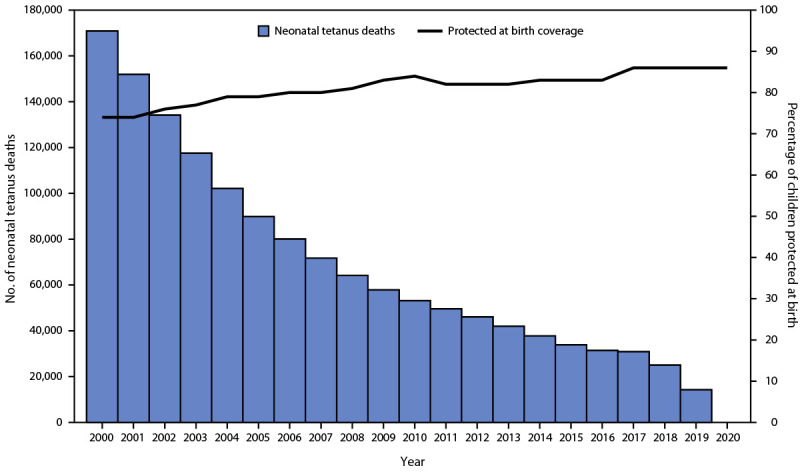
Estimated number of neonatal tetanus deaths* and estimated proportion of children protected at birth^†^ against tetanus — worldwide, 2000–2020^§^ **Sources: **Neonatal tetanus data: WHO Global Health Observatory Data Repository (2000–2018) and the Global Health Data Exchange (2019), Protected at birth data: WHO/UNICEF Joint Reporting Form on Immunization (2000–2020). **Abbreviations:** TTCV = tetanus toxoid–containing vaccine; WHO = World Health Organization. * The number of deaths is estimated from mathematical models that compute the yearly incidence and mortality for each country using the baseline rate of neonatal tetanus before introduction of TTCVs and promotion of clean deliveries, with adjustment for the estimated proportion of women vaccinated with TTCV and deliveries assisted by trained personnel. ^†^ The status of an infant born to a mother who received 2 doses of TTCV during the last birth, ≥2 doses with the last dose received ≤3 years before the last delivery, ≥3 doses with the last dose received ≤5 years earlier, ≥4 doses with the last dose received ≤10 years earlier, or receipt of ≥5 previous doses. ^§ ^Data on deaths for 2020 were not available.

Most neonatal tetanus deaths occur in remote communities, which leads to underreporting. Hence, mathematical models are used to better estimate the number of neonatal tetanus deaths (*9*). The estimated number of neonatal tetanus deaths decreased by 92% from 170,829 (2000) to 14,230 (2019) (Figure 2). In 2019, tetanus accounted for 0.4% of all neonatal deaths, a decrease from 7% in 2000.

## Validation of Maternal and Neonatal Tetanus Elimination

When a country believes it has eliminated MNT, validation activities are implemented, consisting of review of district-level core indicators, including reported neonatal tetanus cases per 1,000 live births and review of the surveillance system, percentage of clean deliveries assisted by a skilled birth attendant, and TTCV2+ coverage among pregnant women (*6*); the country also uses supplementary indicators, including TTCV SIA coverage, antenatal care coverage,**** infant coverage with 3 doses of the diphtheria, tetanus, and pertussis (DTP) vaccine, socioeconomic indices, urban versus rural status, field visits to assess the performance of the health system, validation surveys of poorly performing districts, and assessment of long-term plans for sustaining elimination.^††††^ During 2000–2020, 47 (80%) of 59 priority countries were validated to have achieved MNTE, and 12 remain to be validated (Table) (Figure 1). In addition, by 2020, three countries were partially validated to have achieved elimination in some regions: Mali (Southern regions), Nigeria (Southeast and Southwest zones), and Pakistan (Punjab province).^§§§§^

## Sustainability of Maternal and Neonatal Tetanus Elimination

Once countries are validated for MNTE, WHO recommends four strategies to sustain elimination: 1) providing 3 primary doses of DTP during infancy and 3 TTCV booster doses at ages 12–23 months, 4–7 years, and 9–15 years; 2) checking maternal tetanus vaccination status during antenatal care and providing TTCV2+ to pregnant women, if needed, to ensure that ≥70% of infants are protected at birth; 3) promoting ≥60% clean deliveries through increased access to a skilled birth attendant ; and 4) maintaining strong neonatal tetanus surveillance (*6*). After validation, WHO recommends that countries conduct annual neonatal tetanus risk analyses as part of an immunization desk review and complete postvalidation assessments every 5 years to identify whether elimination status is maintained and take corrective actions as needed (*6*). In 2020, 14 (30%) of the 47 priority countries validated for MNTE achieved ≥90%^¶¶¶¶ ^coverage with 3 doses of DTP; TTCV booster doses***** were provided to children aged 12–23 months in 11 (23%) of those countries, to children aged 4–7 years in 12 (26%) countries, and to children aged 9–15 years in nine (19%) countries. In 45 (96%) countries, ≥70% of infants were protected at birth against tetanus; and in 34 (72%), ≥60% of births were assisted by a skilled birth attendant.

Five countries (Algeria, Cameroon, Djibouti, Indonesia, and Timor-Leste) implemented postvalidation assessments for corrective actions and have met the sustainability indicators for infants protected at birth and the percentage of births with access to a skilled birth attendant. In addition, Cameroon conducted annual neonatal tetanus risk analyses and used assessment outcomes for corrective action by targeting women of reproductive age in high-risk districts with two rounds of TTCV SIAs to sustain MNTE.

## Discussion

Substantial progress has been made toward global MNTE; 80% of the 59 priority countries were validated to have achieved MNTE by the end of 2020. Progress can be attributed to increases in TTCV2+ coverage among women of reproductive age in 34 (58%) of 59 priority countries, implementation of intensive SIAs in high-risk districts, and a 30% increase in deliveries with a skilled birth attendant. These efforts contributed to a 16% increase in infants protected against tetanus at birth and a 92% decline in estimated neonatal tetanus mortality since 2000.

Although progress has been made, countries that have not achieved MNTE still face several challenges. First, suboptimal health systems, evidenced by low vaccination coverage and low proportions of safe and clean deliveries assisted by a skilled birth attendant, make it difficult to adequately implement MNTE strategies. Second, conflict and political instability in some countries contribute to districts remaining inaccessible and at high risk for the incidence of maternal and neonatal tetanus. Lastly, country immunization programs might have competing priorities in addressing the overall incidence of vaccine preventable diseases (e.g., measles and polio) or responding to outbreaks (e.g., Ebola and COVID-19) that hinder their ability to achieve MNTE. During 2020, the COVID-19 pandemic affected TTCV SIAs planned in five countries.

Complete eradication of tetanus is not possible because tetanus spores are ubiquitous in the environment. Therefore, countries need to implement strategies to sustain MNTE. Only five of 47 countries validated for MNTE have conducted the recommended postvalidation assessments, and only 12 have introduced ≥1 TTCV booster doses in their routine immunization schedule. This low uptake could be attributed to competing priorities and the deprioritizing of MNTE once countries are validated, which put countries at risk for reemergence of MNT (*6*). Combining MNTE postvalidation assessments with review of immunization programs and integrating childhood and adolescent tetanus vaccination with other immunization activities (e.g., measles vaccination during second year of life, school vaccination programs, or human papillomavirus vaccination) promote better efficiency and use of resources and help sustain MNTE. Neonatal tetanus case-based surveillance could also be integrated into polio and measles case-based surveillance; community engagement might help raise awareness of neonatal tetanus and serve to strengthen community-based vaccine preventable disease surveillance systems (*8*).

The findings in this report are subject to at least three limitations. First, TTCV coverage among pregnant women can underestimate true tetanus protection because it excludes women who were unvaccinated during current pregnancy but protected through previous vaccination or those missing documentation of previous doses (*6*). Second, the percentage of infants protected at birth could be underestimated because of doses provided outside routine services (*6*). Finally, <10% of neonatal tetanus cases and deaths are estimated to be reported (*2*); although neonatal deaths are projected using mathematical models, cases and deaths might still be underestimated, especially in communities with suboptimal health systems.

The Immunization Agenda 2030,^†††††^ the global immunization strategy for the next decade, includes MNTE as an endorsed vaccine-preventable disease elimination target. To achieve and sustain MNTE, strong national commitment and integration are needed, including integrating MNTE activities with polio, measles, cholera, yellow fever, or other vaccine-preventable disease SIAs, using MNTE to promote equitable access to health services, such as clean deliveries, and promoting a life course approach to tetanus vaccination by integrating TTCV booster doses in school health programs and other life course immunization platforms (*10*).

**Summary**

**What is already known about this topic?**

In 1999, the maternal and neonatal tetanus (MNT) initiative was relaunched to focus on 59 priority countries still at risk for maternal and neonatal tetanus.

**What is added by this report?**

During 2000–2020, 47 countries achieved elimination of MNT, reported neonatal tetanus cases decreased 88%, and estimated deaths declined 92%. Despite progress, 12 countries have not achieved elimination and are challenged by conflict, insecurity, and competing priorities. Other countries are struggling to maintain elimination.

**What are the implications for public health practice?**

To achieve MNT elimination in remaining priority countries and to maintain it globally, efforts are needed to enhance routine vaccination, integrate tetanus activities with other health activities, and promote a life-course vaccination approach for tetanus protection.

## Footnotes

* Tetanus occurring during pregnancy or within 6 weeks of the end of pregnancy; maternal tetanus infection occurs during abortion, miscarriages, or birth with unhygienic delivery. Neonatal tetanus occurs during the first 28 days of life, either following the cutting of the umbilical cord under nonsterile conditions or applying nonsterile traditional remedies to the umbilical stump in an infant without passively (transplacentally) acquired maternal antibodies.

† The occurrence of less than one neonatal tetanus case per 1,000 live births per year in every district in every country.

§ Neonatal tetanus elimination is considered a proxy for maternal tetanus elimination; the same strategies for elimination are shared.

¶ Initially, the total number of priority countries was 57. The creation of Timor-Leste in 2002 and South Sudan in 2011 increased the number of priority countries to 59.

** Tetanus toxoid (TT2+) or tetanus-diphtheria toxoid (Td2+).

†† Mass vaccination campaigns that aim to administer doses of tetanus toxoid–containing vaccines to women of reproductive age.

§§ Districts considered at high risk because the estimated neonatal tetanus case rate exceeds one per 1,000 live births, clean delivery coverage is <70%, and coverage with 3 tetanus toxoid–containing vaccine doses among pregnant women is <80% during the past 5 years.

¶¶ A doctor, nurse, midwife, or health worker trained in providing lifesaving obstetric care, including giving necessary supervision, care, and advice to women during pregnancy, childbirth, and the postpartum period.

*** The status of an infant born to a mother who received 2 doses of TTCV during the last birth, ≥2 doses with the last dose received ≤3 years before the last delivery, ≥3 doses with the last dose received ≤5 years earlier, ≥4 doses with the last dose received ≤10 years earlier, or receipt of ≥5 previous doses.

††† Neonatal mortality data were unavailable for 2020. http://ghdx.healthdata.org/gbd-results-tool

§§§ A postvalidation assessment comprises a review of data to determine whether MNTE indicator standards are being maintained and to identify districts potentially at risk of not sustaining MNTE. Postvalidation assessments include field visits and interviews at both the facility and community level, cross checking the reported coverage of tetanus toxoid–containing vaccines, antenatal care, and skilled birth delivery. The assessment also includes bottleneck analysis and development of a work plan and time frame for implementing corrective actions, if needed.

¶¶¶
https://immunizationdata.who.int/pages/incidence/ttetanus.html?CODE = Global&DISEASE = NTETANUS&YEAR =

**** Antenatal care coverage is the percentage of females aged 15–49 years with a live birth who received antenatal care provided by skilled health personnel (doctor, nurse, or midwife) at least once during pregnancy.

††††
https://www.who.int/immunization/documents/MNTE_Validation_survey_WHO_IVB_18.15.pdf

§§§§
https://www.who.int/initiatives/maternal-and-neonatal-tetanus-elimination-(mnte)/the-partnership

¶¶¶¶
https://www.who.int/data/gho/data/indicators/indicator-details/GHO/diphtheria-tetanus-toxoid-and-pertussis-(dtp3)-immunization-coverage-among-1-year-olds-(-)

*****
https://immunizationdata.who.int/pages/schedule-by-disease/tetanus.html?TARGETPOP_GENERAL = GENERAL
